# Degradation of chemical alarm cues and assessment of risk throughout the day

**DOI:** 10.1002/ece3.760

**Published:** 2013-09-17

**Authors:** Douglas P Chivers, Danielle L Dixson, James R White, Mark I McCormick, Maud C O Ferrari

**Affiliations:** 1 Department of Biology, University of SaskatchewanSaskatoon, SasKatchewan, S7N 5E2, Canada; 2 ARC Centre of Excellence for Coral Reef Studies and School of Marine and Tropical Biology, James Cook UniversityTownsville, Queensland, 4811, Australia; 3 Department of Biomedical Sciences, WCVM, University of SaskatchewanSaskatoon, SasKatchewan, S7W 5B4, Canada

**Keywords:** Alarm cues, coral reefs, information use, predator–prey interactions, risk assessment.

## Abstract

The use of chemical information in assessment of predation risk is pervasive across animal taxa. However, by its very nature, chemical information can be temporally unreliable. Chemical cues persist for some period of time after they are released into the environment. Yet, we know surprisingly little about the rate of degradation of chemical cues under natural conditions and hence little about how they function in temporal risk assessment under natural conditions. Here, we conducted an experiment to identify a concentration of fresh alarm cues that evoke a strong antipredator response in coral reef damselfish, *Pomacentrus ambonensis*. We then tested the rate at which these alarm cues degraded under natural conditions in ocean water, paying attention to whether the rate of degradation varied throughout the day and whether the temporal pattern correlated with physicochemical factors that could influence the rate of degradation. Fresh alarm cues released into ocean water evoke strong avoidance responses in juvenile fish, while those aged for 30 min no longer evoke antipredator responses. Fish exposed to cues aged for 10 or 20 min show intermediate avoidance responses. We found a marked temporal pattern of response throughout the day, with much faster degradation in early to mid-afternoon, the time of day when solar radiation, temperature, dissolved oxygen, and pH are nearing their peak. Ecologists have spent considerable effort elucidating the role of chemical information in mediating predator–prey interactions, yet we know almost nothing about the temporal dynamics of risk assessment using chemical information. We are in dire need of additional comparative field experiments on the rate of breakdown of chemical cues, particularly given that global change in UV radiation, temperature, and water chemistry could be altering the rates of degradation and the potential use of this information in risk assessment.

## Introduction

Due to the unforgiving nature of predation, most animals have invested heavily in antipredator defense mechanisms (Crowl and Covich 1990; Lima and Dill 1990; Brönmark and Miner 1992). Morphological defenses, such as protective spines and armor, deter attacks and reduce the probability of capture in a variety of animals (Arnqvist and Johansson 1998; Hoverman et al. 2005). Many prey species have cryptic coloration to avoid detection or alternatively are brightly colored advertising noxious or toxic properties to would-be predators (Cuthill et al. 2005; Stankowich et al. 2011). Behavioral defenses also limit the success of predators, with prey avoiding specific locations and/or limiting their activity during times of day that predators are hunting (Lima 1998; Ferrari et al. 2009). When prey do encounter predators, they can also flee or hide to escape an attack (Sih 2005).

One of the prerequisites for successful predator avoidance is that prey animals recognize predators or high-risk situations as dangerous (Brown and Chivers 2006). Information that allows prey to recognize risk can come from a variety of sources, including nearby conspecifics (Griffin 2004; Crane and Ferrari 2013). Prey may respond to the visible flight responses or alarm calls of conspecifics (Blumstein and Armitage 1997). They may also respond to chemical cues released by nearby individuals that have detected a predator (often referred to as disturbance cues) or to chemical cues released by prey that have been attacked by predators (often referred to as damage-released alarm cues) (Chivers and Smith 1998; Ferrari et al. 2010). Both of these chemical sources of information could provide prey with an early warning of a potential attack. Indeed, several studies have shown that this early warning increases the probability of survival during a staged encounter with a predator (Hews 1988; Mathis and Smith 1993a; Mirza and Chivers 2001).

Damage-released alarm cues are common in freshwater and marine organisms and known to induce adaptive changes in morphology in prey animals (Stabell and Lwin 1997; Chivers et al. 2008) and are crucial in facilitating learned recognition of predators (Mathis and Smith 1993c; Ferrari et al. 2005) and dangerous habitats (Chivers and Smith 1995). There are many hundreds of studies that have investigated the importance of these cues in mediating predator–prey interactions (Chivers and Smith 1998; Ferrari et al. 2010). It is therefore surprising that we know almost nothing about the temporal aspects of risk assessment using alarm cues (Ferrari et al. 2010). When a prey individual is captured and alarm chemicals are released, the prey detecting the chemical cues are aware that a nearby individual was *recently* captured. However, to understand the value of this information, we need to begin to consider what *recently* actually means. In systems where predators have the ability to eat multiple prey in a short time, an alarm cue released 1 min ago would probably be important as a risk assessment cue. What about an alarm cue released 10 min ago or one released an hour or even a day ago? How long do the chemicals actually persist in the environment? Short-lived chemicals could provide very accurate temporal information about risk. In contrast, those cues that last for many hours would be much less temporally reliable, but nonetheless would provide at least some information.

Three studies have investigated these sorts of temporal risk assessment questions using chemical alarm cues. In a laboratory experiment, Hazlett (1999) showed that alarm cues of crayfish (*Orconectes virilis)* can persist (i.e., be detectable by conspecifics) for more than 6 h. Likewise, Wisenden et al. (2009) demonstrated that alarm cues of freshwater fish (fathead minnows, *Pimephales promelas*) and amphipods (*Gammaruslacustris*) may last at least 3 h, but not more than 6 h. In these experiments, the alarm cues were prepared in the laboratory with clean dechlorinated tap water. We caution that such designs need to be interpreted carefully because the absence of sunlight could influence photodegradation of the alarm chemicals and the absence of appropriate biofauna could influence the rate of biodegradation. Wisenden et al. (2009) attempted to use a natural trapping experiment to confirm their laboratory findings, but the results of their experiment were somewhat ambiguous. Ferrari et al. (2007b) tested the rate of breakdown of wood frog tadpole alarm cues in a natural pond and found that tadpoles responded to cues released 5 min following injection into the pond, but did not respond to the same cue after 2 h. Clearly, there is a dire need for experiments designed to determine the rate of breakdown of alarm cues to understand the role of the cues in mediating predator–prey interactions. The goal of our current work was to determine the duration that alarm cues of juvenile damselfishes (*Pomacentrus amboinensis)* persist under natural conditions around Lizard Island in the Great Barrier Reef. Several studies have recently shown the importance of alarm cues in risk assessment in coral reef damselfishes (McCormick and Larson 2008; Ferrari et al. 2011; Lonnstedt et al. 2013; Chivers et al. in press).

Here, we used a two-channel choice flume to assess avoidance behavior of damselfish to the alarm cues of conspecifics. First, we identified a concentration of fresh alarm cues that consistently lead to a strong avoidance response. We then prepared additional batches of fresh alarm cues and introduced them into ambient seawater held in floating containers in the ocean where they were exposed to a natural temperature and light regime. The containers also held a natural sand substrate to ensure that the water was in contact with natural substrate-born biofauna that could breakdown the alarm cues. For each container, we sampled the water immediately after placing the alarm cues into the container and at 10, 20, and 30 min post-injection. We used the choice flume to determine whether the cues remained active after various amounts of time had elapsed. The breakdown of alarm cues could be influenced by both photodegradation and biodegradation, the rates of which could vary based on abiotic conditions including temperature, pH, dissolved oxygen, and solar radiation. Consequently, we replicated the start time of the experiment to test whether the rate of degradation varied throughout the day and could be correlated with any abiotic factors.

## Methods

### Fish collection and alarm cue preparation

Our experiment took place at the Lizard Island Research Station (14°40′S, 145°28′E), on the Great Barrier Reef, Australia, in December 2010. Juvenile *P. amboinensis* (16–21 days old) were caught overnight using light traps (Meekan et al. 2001) moored approximately 100 m off the fringing reef. These traps collect fish at the end of their pelagic phase, immediately prior to their settlement to the reef. Fishes caught in the traps were brought back to the station just after dawn and sorted by species, and small groups of *P. amboinensis* were transferred into 35-L aquaria, where they were fed three times a day with live *Artemia nauplii*.

Alarm cues were prepared by euthanizing donor fish by cold shock and then making a series of vertical cuts along both sides of each fish. Afterward, the fish were rinsed in 60 mL of seawater, and the resulting solution was added to a plastic pail containing 16 liters of water. Our initial experiment tried to identify the number of cuts required to evoke a strong antipredator response in our test fish. We used a total of 6 donor fish, each of which had 4, 6, 8, 10, or 12 cuts per side. This resulted in a total of 48, 72, 96, 120, or 144 cuts added to the 16 liters of water. The field of chemical ecology suffers from not having a good understanding of the chemistry of alarm cues or information on the amount of alarm cues that are released during a predator attack (Ferrari et al. 2010). Therefore, it is somewhat difficult for us to know whether the amount of alarm cues released during a predator attack matches the concentrations we used in our experiment. Depending on the relative size of the predator and prey, and the size of the predator's mouth, the prey may take several minutes to manipulate and consume the prey (Chivers et al. 1996; Ferrari et al. 2007a). In such a case, there is likely more tissue damage than that in the high concentration treatment we used in our experiment (Ferrari et al. 2007a). However, in other cases, the prey may be swallowed with little damage.

### Behavioral assay

Our behavioral assay was a slight modification of the methods of Dixson et al. (2010), in which fish were tested using a two-channel choice flume (13 × 4 cm). The flume had a constant gravity-driven flow of 100 mL min^−1^ per channel throughout all trials. Flow rates were measured using a flow meter, and a dye test ensured that the 2 channels exhibited distinct and parallel water flow, with no turbulence or eddies. Prior to each trial, individual fish were isolated in 100-mL jars and left to acclimate for 10 min. An individual fish was placed into the center of the downstream end of the choice flume and acclimated to the two water choices for 2 min. At the end of the acclimation period, the position of the fish on either side of the chamber was recorded at 5-sec intervals for 2 min. The side of the flume containing the treated and untreated ocean water was alternated to ensure that a side preference was not be displayed by individuals. In each trial, the larva was given a choice in the flume chamber between a water source (ocean water) treated with alarm cues and an identical water source without that cue. It is well established that fish avoid the side of the flume when they detect risk (Munday et al. 2010). Fish used in the experiment were randomly selected from the holding tanks. Each fish was tested only once.

### Experiment 1

Prior to determining the rate of degradation of chemical alarm cues, it was critical to identify a concentration of fresh alarm cues that provided a consistently high-level antipredator response in our test apparatus. Variation from this consistently high-level response could then be used as a sensitive degradation assay. Consequently, for experiment 1, we prepared 5 alarm cue solutions that varied over a threefold difference in concentration (see alarm cue preparation above for details). We conducted a total of five trials in each of the five alarm cue concentration treatments along with five fish in the blank control treatment where ocean water was provided in both channels of the flume.

### Experiment 2

The goal of this experiment was to (1) quantify the rate of degradation of chemical alarm cues under natural conditions and (2) to identify whether the rate of degradation varied throughout the day and whether the rate of breakdown of the cues correlated with any abiotic parameters we measured. We prepared multiple batches of fresh alarm cues (144 cuts in 60 mL of seawater) and introduced each batch into a 16-L bucket that was floating in the ocean and exposed to ambient temperature and photoperiod. The buckets were held in approximately 1 m of water using floats. Each bucket contained approximately 3 cm of natural coral sand substrate that was collected at a water depth of 1 m.

**Figure 1:**
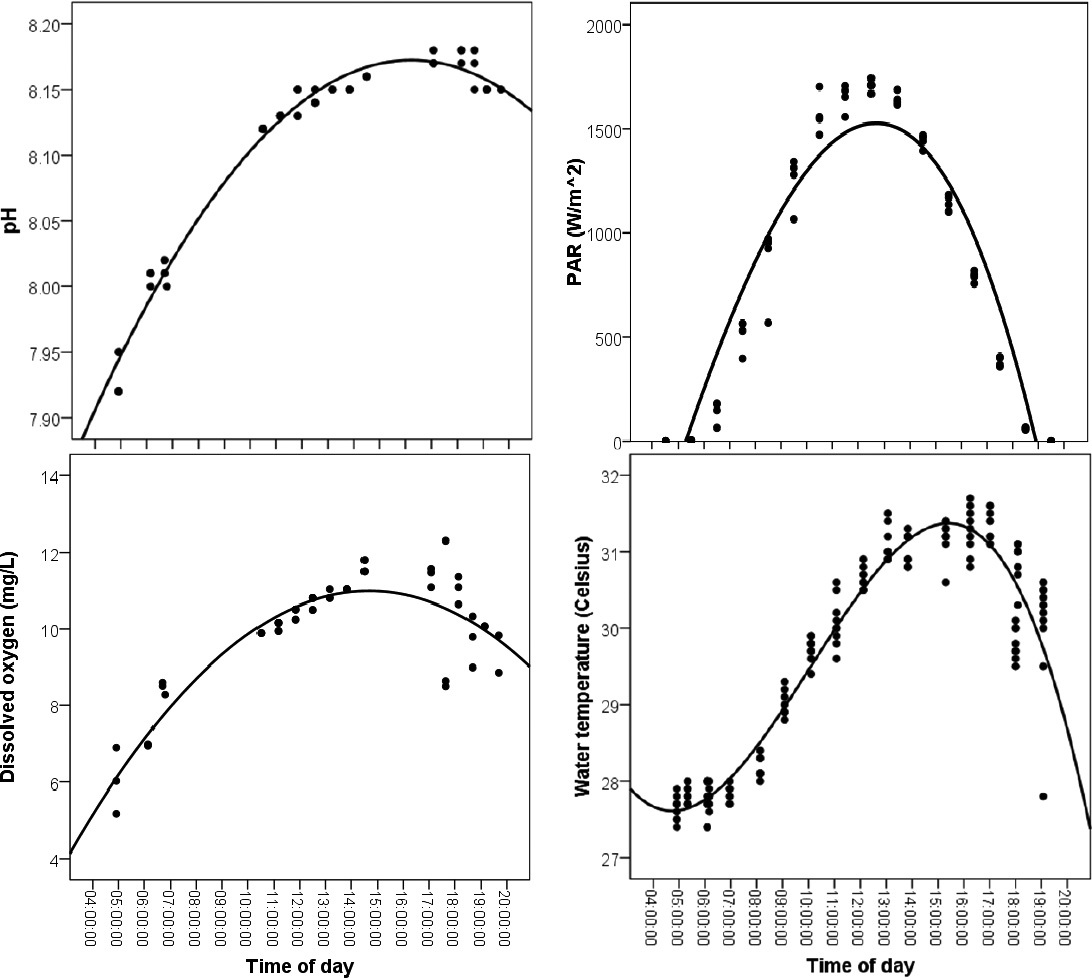
Measurements of physiochemical parameters (temperature, pH, and dissolved oxygen) that were taken during the experiment. The PAR profile was from the exact same date 1 year later.

Our protocol consisted of adding the alarm cue solution to the bucket and gently stirring the water to ensure that the stimulus was evenly dispersed. We then immediately removed approximately 2 liters of water for behavioral trials and immediately conducted the avoidance experiment. Water was also removed at 10, 20, and 30 min post-injection. For each of the four time intervals (0, 10, 20, and 30 min after injection), the water was used to run four replicate behavioral trials for a total of 16 fish tested per bucket. Fish were only used once. By comparing the intensity of behavioral response of the fish at the four time points, we were able to assess the rate at which the alarm cues breakdown, if indeed the cues breakdown over this time interval. To facilitate rapid testing of the fish, we employed two flumes. The rate of breakdown could be highly variable throughout the day depending on factors such as temperature, pH, dissolved oxygen, and solar radiation. Consequently, we initiated 18 replicate buckets over the course of the day beginning at 0500 and ending at 1900. The experiment was run over 4 consecutive days. We recorded temperature, pH, and dissolved oxygen during most of the trials (Fig. 1), but did not have access to a photometer and hence did not have the ability to determine the specific level of solar radiation for each trial. However, all days were free of all but sporadic cloud cover; hence, the peak in solar radiation corresponds with afternoon sun. The sun rose at approximately 0545 and set at 1845 h each day. To obtain a profile of sunlight exposure at this time of year, we obtained photosynthetically active radiation (PAR) data from the following year (same dates) from the Great Barrier Reef Ocean Observing System (courtesy of AIMS). Data were not available for 2010. While the data represent a different year, the sun rises, sets, and peaks at the same time, hence providing valuable information as to the radiation profile experienced during our experiment. The experimental testing occurred outdoors in a shaded location to reduce the possibility of breakdown of chemical cues once the subsample of water had been removed from the pail for testing.

### Statistical analyses

#### Experiment 1

For each of the alarm cue concentrations (0, 48, 72, 96, 120, or 144 cuts/16 L), we used a one-sample *t*-test to compare the percent of time fish spent in the alarm cue arm of the flume to a random choice (50%).

We then compared among the six concentrations using a one-way ANOVA, followed by Tukey's post hoc tests. Testing a variety of concentrations allows us to identify a concentration of fresh alarm cues that evoked a strong antipredator response in our test apparatus, but also allowed us to examine the shape of the degradation curve.

#### Experiment 2

We used a one-way ANOVA to compare the percentage of time fish spent in the alarm cue arm of the flume at each of the 4 time intervals following introduction of alarm cues in the pail (0, 10, 20, and 30 min after introduction). To further explore the pattern of degradation throughout the day, we used a curve fitting option to best describe the relation between alarm cue avoidance and time of day for cues that had aged different periods of time (0, 10, 20, or 30 min).

## Results

### Experiment 1

**Figure 2:**
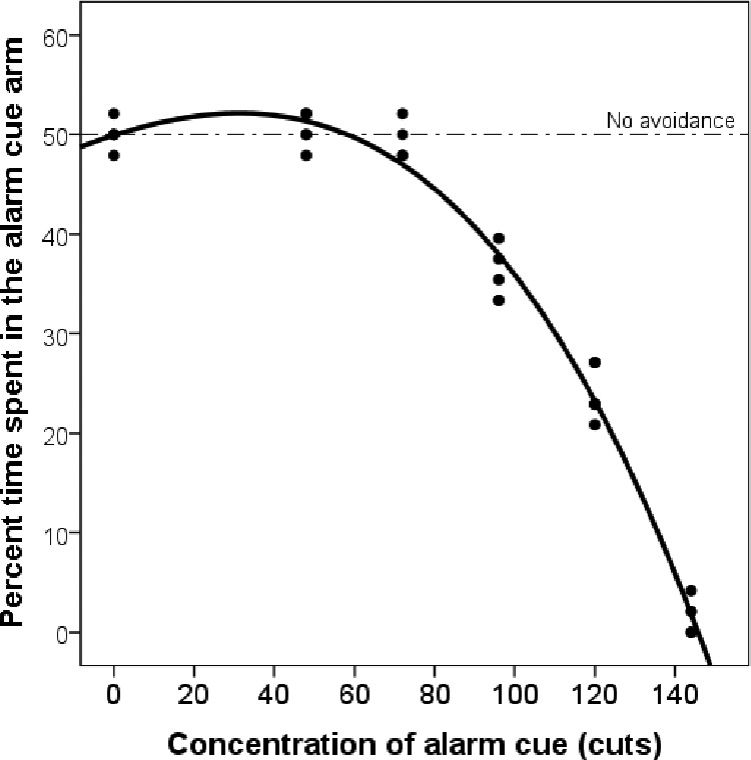
Mean (±SE) proportion of time damselfish spent in the alarm cue arm of the flume when exposed to various concentrations of alarm cues in experiment 1 (*n* = 5/treatment).

A series of one-sample t-tests revealed that there was no significant avoidance of the 0 cut (*t*_4_ = 0.0, *P* > 0.95), 48 cut (*t*_4_ = 5.4, *P* = 0.62), or 72 cut (*t*_4_ = −1.0, *P* = 0.37) treatments, but there was significant avoidance at each of the three higher concentrations (96 cuts: *t*_4_ = 11.7, *P* < 0.001; 120 cuts: *t*_4_ = 20.7, *P* < 0.001 and 144 cuts: *t*_4_ = 62.01, *P* < 0.001, Fig. 2). The ANOVA revealed a significant difference between treatments (*F*_5,24_ = 422, *P* < 0.001) with a clear gradation among the various concentrations. Tukey's tests showed that fish in the 0, 48, and 72 cut treatments showed similar avoidance responses, but as the concentration of cues increased at each subsequent concentration, there was significantly greater avoidance of the cue (all *P*'s < 0.05).

### Experiment 2

**Figure 3:**
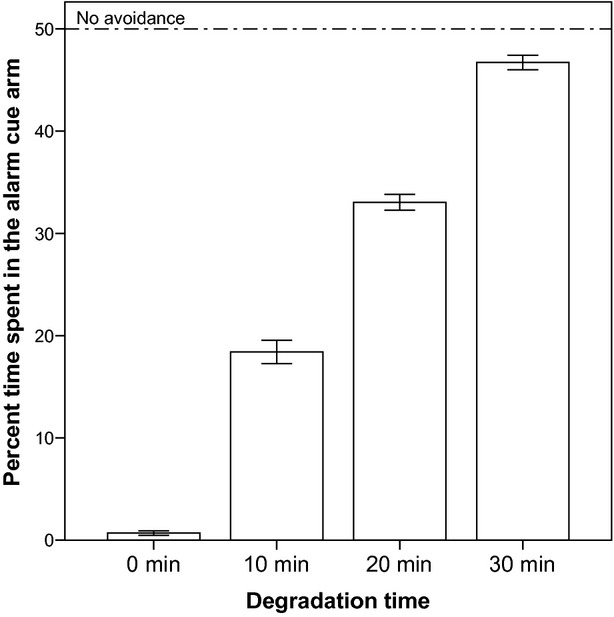
Mean (±SE) proportion of time damselfish spent in the alarm cue arm of the flume in experiment 2. Experiments were undertaken when the alarm cues had aged in ocean water for 0, 10, 20, or 30 min (*n* = 70–72 /degradation time).

**Figure 4:**
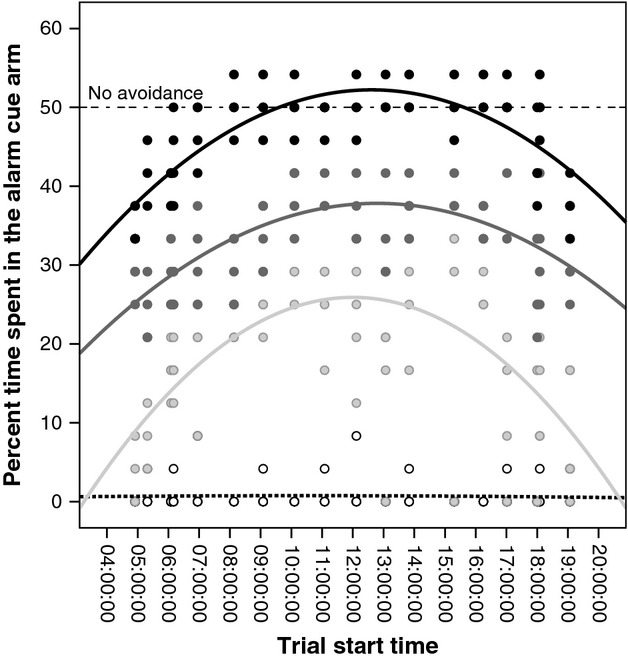
Proportion of time damselfish spent in the alarm cue arm of the flume at different times of day with cues that aged for different periods of time. Different color dots and lines correspond with cues that aged different periods of time. The darker the dots and lines, the longer the cues had aged. For cues that aged 10, 20, or 30 min, the relationship between avoidance and time of day was best described by a quadratic curve (*n* = 4/time of day/degradation time).

The ANOVA revealed a significant effect of degradation time on the response of the fish to the alarm cue (*F*_3,281_ = 639, *P* < 0.001, Fig. 3). Fish exposed to fresh alarm cues showed nearly 100% avoidance of the alarm cues. Fish exhibited a significantly greater reduction in their avoidance at each subsequent time interval (Tukey's post hoc test: all *P* < 0.001). While all fish decreased their avoidance of the alarm cue (AC) side of the flume as the cues aged, we noted a striking effect of time of day on the responses. For each degradation time (10, 20, and 30 min), the relationship between avoidance and time of day was best described by a quadratic curve (see Fig. 4). In early to mid-afternoon, there was a faster rate of degradation than at other times of day. This time of day matches the time when temperature, pH, dissolved oxygen, and solar radiation are reaching their peak, and hence, one or all of these factors could be contributing to the pattern observed (Fig. 1).

**Figure 5:**
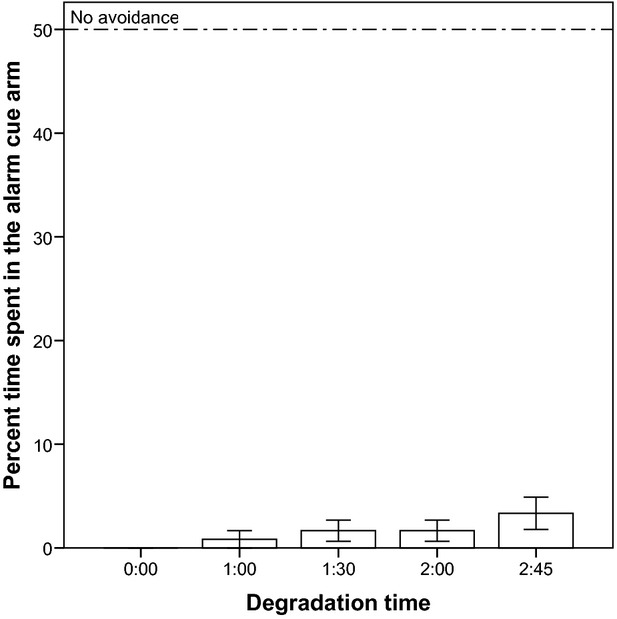
Mean (±SE) proportion of time damselfish spent in the alarm cue arm of the flume when alarm cues were degraded for different periods of time (hours) in the laboratory (temperature: 25.8 C) (*n* = 6/degradation time).

Without additional manipulative experiments, it is not possible to attribute the rate of degradation to any one abiotic factor; however, the magnitudes of the change in temperature and dissolved oxygen we observed were relatively small. The magnitude of the change in pH was also small, but less so given that pH is measured on a log scale. We have preliminary trials indicating that alarm cues placed into buckets containing seawater and sand substrates and held at 25.8°C (pH 8.14–8.15) do not breakdown for at least 3 h in the laboratory under artificial lighting (Fig. 5). This is vastly different from what happens in the field. These points lead us to speculate that solar radiation may be primarily responsible for the degradation. We provide some tests of the role of solar radiation in the following section, but caution that additional manipulative experiments are needed.

We tested whether or not solar radiation would affect the speed at which the fish would stop avoiding the alarm cue using a 2 x 4 ANOVA. We did not have a photometer to test different levels of radiation, so we designated all trials occurring between 0700 and 1800 as trials with a significant amount of solar radiation and those occurring before or after this time as trials with limited solar radiation. We did not use sunrise and sunset as our cutoff because the angle of the sun and local topography were such that light did not reach the pails early in the morning or late in the evening. There was a significant interaction between the amount of solar radiation and cue age on the responses of damselfish to alarm cues (*F*_3,277_ = 12.3, *P* < 0.001). This was driven by the fact that the responses of fish in the presence and absence of a significant amount of solar radiation were the same at time 0 (*F*_1,70_ = 0.13, *P* = 0.72), before radiation had a chance to have an effect, but were not the same when the cues had aged 10, 20, or 30 min. Comparing the responses of fish at 10, 20, and 30 min revealed a significant effect of solar radiation (*F*_1,207_ = 108.1, *P* < 0.001), and an effect of cue age (*F*_2,207_ = 362.5, *P* < 0.001), but no cue age x solar radiation interaction (*F*_2,207_ = 1.01, *P* = 0. 37). Post hoc tests on cue age revealed that all 3 times differed from each other (all *P* < 0.001). After 30 min, the response of the fish tested in the presence of solar radiation did not differ from random (one sample *t*-test, *t*_43_ = 0.0, *P* > 0.99). However, the fish tested in the absence of solar radiation still showed a significant avoidance of the alarm cue (one sample *t*-test, *t*_26_ = −7.63, *P* < 0.001 – Fig. 3).

## Discussion

The results of our study highlight that chemical alarm cues of coral reef damselfish degrade rather quickly under natural conditions. In our bioassay, we found nearly 100% avoidance of freshly prepared alarm cues. However, after the cues aged for only 30 min, we observed little avoidance. The responses for cues aged 10 and 20 min were intermediate. What was most striking was that we observed very different patterns of responses to aged cues at different times of the day. Given that we failed to find a temporal effect on the response of fish to fresh cues, we concluded that the differential responses to aged cues were driven by the aging of the cues, rather than a diel change in the fish's response. Aged cues failed to evoke antipredator responses at midday, but they did both early and late in the day. We speculate that this corresponds with the highest rate of breakdown of alarm cues occurring in the afternoon.

The relatively short active time for alarm cues that we documented contrasts an extensive literature showing that prey animals frequently respond to odors of predators fed conspecifics of the prey, but not to odors of predators fed a different diet (Mathis and Smith 1993c; Chivers and Mirza 2001). Indeed, one study showed that minnows (*Pimephales promelas*) responded to odors of predators fed minnows that have alarm cues, but did not respond to predators fed minnows that lacked alarm cues (Mathis and Smith 1993b). In predator-diet studies, predators are fed specific diets for days and then not fed for a day or two before odor cues are collected. The fact that odors that are days old can evoke antipredator responses implies that alarm cues survive digestion for days in the predators' gut or that the breakdown products of the alarm cues can last for days.

Our work provides some evidence that the rate of breakdown of alarm cues is dependent on solar radiation. Indeed, early in the day and late in the evening when the sun was not shining directly into the pails, the rate of alarm cue breakdown was significantly lower. This raises the interesting question of whether fishes can gain different temporal information from chemical cues depending on time of day and ambient weather conditions. Do chemical cues last longer on cloudy days? Do alarm cues released near the surface have a shorter half-life than those released at a depth where solar penetration is reduced? The specific wavelengths of light that could be responsible for the photodegradation are unknown to us, but UV radiation is known to cause the breakdown of many organic molecules (Hays et al. 1996). If this is the case, then any factor that influences the level of UV radiation will alter the rate of degradation. Stratospheric ozone depletion, a major environmental concern, particularly in the Southern Hemisphere (Smith et al. 1992), could lead to increased rates of alarm cue degradation, while the addition of turbidity and dissolved organic carbon associated with anthropogenic change (Wenger and McCormick, 2013) could lead to decreased rates of degradation.

We must be cautious in our conclusion that solar radiation is primarily responsible for differences in the rate of degradation of alarm cues. Clearly, additional manipulative experiments are in order. Temperature, pH, and dissolved oxygen followed the same general temporal pattern as solar radiation with peaks in the mid-afternoon, and hence, these factors could be responsible for the effects we observed. We had a relatively narrow window of temperatures, pH, and dissolved oxygen in our study; hence, these factors seem much less plausible than solar radiation. However, other prey species in other systems do experience extreme changes in physicochemical conditions. For example, temperate fishes frequently experience greater than a 20°C change in temperature throughout the year. If temperature is a factor mediating the rate of alarm cue breakdown, then we could easily imagine seasonal differences in temporal information use. The IPCC predicts a 3°C increase in ocean temperatures by the end of the century (IPCC 2007). It seems plausible that such warming could increase the rate of alarm cue degradation and alter chemosensory risk assessment, even for fishes in warm tropical waters. The acidity of our oceans is also predicted to change considerably over the next century (Kleypas et al. 2006). If alarm cue breakdown is linked to ocean pH, then we should expect to see the opposite effect as with an increase in temperature; ocean acidification should reduce degradation of alarm cues. This is based on the observation in our study that there is a higher rate of breakdown in mid-afternoon when pH is the highest.

We are in dire need of comparative field experiments designed to test the rate of breakdown of chemical cues that indicate risk. Besides the work of Ferrari et al. (2007b) and Wisenden et al. (2009) examining the rate of breakdown of alarm cues in freshwater ponds and lakes, we have little information in other systems. However, there are a few other studies that have attempted to determine temporal aspects of risk assessment using predator odors. Bytheway et al. (2013) recently showed that rats (*Rattus fuscipes*) avoid fresh dog (*Canis lupus familiaris*) scent, but fail to respond to dog scent that was aged for two days. Peacor (2006) found that the time period that bullfrogs (*Ranacates beiana*) responded to odors of larval dragonflies (*Anax junius*) was in the order of 2–4 days. Interestingly, the length of time was shorter when the dragonfly cue was aged in pond water compared with when it was aged in well water. In a similar experiment, Fraker (2009) concluded that greenfrog (*Rana clamitans*) tadpoles lacked the perceptive ability to reliably assess the age of predator odors. Tadpoles responded to dragonfly cues aged up to 48 h but not 72 h. He argues that tadpoles overemphasize risk resulting in a disproportionately strong antipredator response. Unfortunately, Fraker (2009) used well water not pond water, and like Peacor (2006), he aged the cues in the laboratory not under natural solar conditions. Our work here suggests that we should use caution in interpreting results of laboratory-based experiments. In the absence of solar radiation and natural biofauna that could breakdown the cues, the rate of degradation could be misleading. In fact, when we conducted preliminary trials to establish the timeframe and concentrations to use in these experiments, we found that the cues prepared in fresh ocean water and held indoors, in pails containing sand substrates, could last for over 3 h in the laboratory. This is vastly different from what we observed under natural conditions.

When a prey animal is captured by a predator and alarm cues are released, the cues not only breakdown, but also become dispersed due to water movements, etc. Our first experiment provided a clear indication of a threat-sensitive response to varying concentrations of alarm cues. As the concentration of cues increased, we saw greater avoidance of the cue. This work is in accordance with several studies that have demonstrated similar concentration effects in other systems (Ferrari et al. 2005). The challenge for researchers that want to understand the importance of chemical cues as information sources will be to understand how prey integrate information about degradation and cue dispersal to make informed decisions. Indeed, there is another intriguing possibility that may also come into play. Are prey able to determine the age of chemical cues irrespective of dilution and degradation? If this were the case, we should expect that the shape of the degradation curve would be different than the shape of the dilution curve. We did not have a consistent degradation curve in our experiment; the shape of the curve (i.e., the rate at which the fish quit responding to the cues) was dependent on time of day. This result is consistent with the possibility that fish can determine the age of chemical information irrespective of degradation and dilution. This hypothesis would be much easier to address if we knew the chemical identity of the alarm cues, but to date, we do not have this information (Ferrari et al. 2010). Bytheway et al. (2013) recently used GC-MS to show that aged predator odors are indeed different than new predator odors and that the difference in the cues may allow rats the ability to age predator cues. Ecologists often think of chemical cues released during predator attacks as long-lasting chemicals that linger in the area. As such, they provide some information about risk but the spatial and temporal aspects of the information are somewhat unreliable. We need to think about the natural rate of breakdown of the cues and the environment in which the cues are dispersing to gain a full appreciation of the spatial and temporal limitations of chemical information sources.
